# Construction Notes

**Published:** 1905-01-07

**Authors:** 


### CONSTRUCTION NOTES.

A NEW ward has been opened at Thames Ditton Cottage Hospital.

AN infectious diseases hospital has been opened at Cashes Green, for Stroud.

A NEW out-patients' department is being added to the Bradford Children's Hospital.

A NEW isolation hospital has been opened at Chesterfield. The cost has been £9,400, or about £293 per bed.

THE foundation stone has been laid of a new dispensary for Nottingham at Hyson Green. The cost is estimated at £3,000.

THROUGH the generosity of Provost Moncur a cancer wing is to be added to the Dundee Royal Victoria Infirmary, costing about £5,500.

THE foundation stone has been laid of a new infirmary for children at Liverpool, which is to take the place of the old premises now pulled down.

